# Factors associated with health care utilization and catastrophic health expenditure among cancer patients in China: Evidence from the China health and retirement longitudinal study

**DOI:** 10.3389/fpubh.2022.943271

**Published:** 2022-11-10

**Authors:** Penghong Deng, Yu Fu, Mingsheng Chen, Lei Si

**Affiliations:** ^1^School of Health Policy and Management, Nanjing Medical University, Nanjing, China; ^2^Center for Global Health, Nanjing Medical University, Nanjing, China; ^3^School of Health Sciences, Western Sydney University, Campbelltown, NSW, Australia; ^4^The George Institute for Global Health, University of New South Wales, Kensington, NSW, Australia

**Keywords:** health care utilization, catastrophic health expenditure, factors, cancer, China

## Abstract

**Background:**

Cancer, the leading cause of mortality in China, is a significant burden on patients, their families, the medical system, and society at large. However, there is minimal data on health service utilization and catastrophic health expenditure (CHE) among cancer patients in China. The objective of this study was to identify factors associated with health care utilization and CHE in Chinese cancer patients.

**Methods:**

The 2018 wave of a nationally representative dataset, the China Health and Retirement Longitudinal Study, was used in our study. Of 18,968 respondents recruited for the analysis, 388 were clinically diagnosed with cancer. CHE was defined as household health expenditure that exceeded 40% of non-food household expenses. A binary logistic regression model was used to identify the risks of cancer exposure among all participants, along with the likelihood of CHE in households with cancer patients at the 40% threshold. A negative binomial regression model was used to identify determinants of health service utilization among cancer patients.

**Results:**

Contracting a family physician (incidence rate ratio IRR: 2.38, 1.18–4.77), Urban Employee Basic Medical Insurance (IRR: 4.02, 1.91–8.46, compared to the uninsured), Urban and Rural Resident Basic Medical Insurance (IRR: 3.08, 1.46–6.49, compared to the uninsured), and higher per-capita household consumption were positively associated with inpatient service utilization. Patients with a college education and above reported a greater number of outpatient visits (IRR: 5.78, 2.56–13.02) but fewer inpatient hospital days (IRR: 0.37, 0.20–0.67). Being diagnosed with a non-cancer chronic non-communicable disease was associated with an increased number of outpatient visits (IRR: 1.20, 1.10–1.31). Of the 388 participants, 50.1% of households had CHE, which was negatively correlated with a larger household size (odds ratio OR: 0.52, 0.32–0.86) and lower socioeconomic status [for quintile 5 (lowest group) OR: 0.32, 0.14–0.72].

**Conclusions:**

The socioeconomic characteristics of cancer patients had a considerable impact on their healthcare utilization. Individualized and targeted strategies for cancer management should be implemented to identify high-risk populations and trace the utilization of care among Chinese cancer patients. Strategic purchasing models in cancer care and social health insurance with expanded benefits packages for cancer patients are crucial to tackling the cancer burden in China.

## Introduction

Cancer is a leading cause of mortality worldwide and a substantial impediment to an optimal expected lifespan (1). There were an estimated 4.5 million new cancer cases and 3 million cancer mortalities in 2020 in China, which with the largest population in the world accounts for the highest percentage of total new cancer cases (~23.7%) and mortalities (30.2%) worldwide (2). Cancer incidence and mortality have been rapidly rising in China (2) due to an aging population and cancer-associated lifestyle behaviors (3). Cancer represents a significant burden on patients, their families, the medical system, and society at large.

Aiming to alleviate the burden of non-communicable diseases including cancer, China initiated comprehensive healthcare reform in 2009 committed to delivering equitable accessibility to primary healthcare services with appropriate quality and financial risk protection for all citizens by strengthening healthcare infrastructure, broadening public health insurance coverage, and reforming the healthcare delivery system (4). The Urban Resident Basic Medical Insurance (URBMI) and New Rural Cooperative Medical Scheme (NRCMS) were merged into the Urban and Rural Resident Basic Medical Insurance (URRBMI) in early 2016, which enhanced the health insurance system's ability to pool financial risks for cancer patients (5). Universal Health Coverage improved the availability and utilization of health services (6), although deficiencies remain in quality, efficiency, spending, and patient satisfaction (4). Cancer patients experienced inequitable access to cancer care due to inequalities in health financing, particularly in rural China (7).

Accessing healthcare services can improve patient health but potentially lead to catastrophic health expenditure (CHE), which we defined as the proportion of out-of-pocket (OOP) spending exceeding 40% of household non-food expenses (8). Delivering high-quality care and protecting families from CHE are widely accepted desirable objectives of the healthcare system. These objectives assume that we understand what health system characteristics benefit patients and the factors that affect health service use and CHE. The demand for cancer care spans from the time of diagnosis to the terminal phase of life, making cancer patients particularly susceptible to CHE. Identifying particularly vulnerable groups *via* susceptibility factors and household characteristics may steer cancer patients to utilize appropriate healthcare services and prevent CHE attributable to cancer care.

Previous studies have highlighted issues related to health service use and economic burden among patients with non-communicable diseases, such as hypertension, diabetes, as well as patients with multiple chronic diseases (9–12). However, factors associated with cancer care utilization and CHE in China remain unclear. Health service use reflects individual behaviors related to obtaining health services to meet their health demands. Existing literature has shown that health service use is affected by individual demographic and socioeconomic factors such as age, gender, marital and employment status, education level, insurance, income (13), and health care system characteristics such as the availability, affordability, and accessibility of drugs and healthcare services (14). Previous studies have reported that health service use among cancer patients differs by sex, age, residence, employment, education, health insurance, household income, tumor site, and tumor stage (15–18). A study of rural-urban disparities among Chinese cancer patients found that rural cancer patients utilized fewer screening and treatment services than urban patients, and that care disparities were significantly influenced by socioeconomic and clinical characteristics (15). Another study on socioeconomic disparities in cancer treatment in China found that a higher proportion of patients with high socioeconomic status underwent surgery and chemotherapy than those with low socioeconomic status (19). Further, socioeconomic status was identified as the most important determinant of treatment modalities for esophageal cancer (16). Evidence from Beijing, China indicated that inpatient costs were 58.6% of total cancer treatment costs, with anti-cancer medication costs accounting for the majority of this burden. Total costs were highly associated with age, tumor type, hospital level, and payment system (20). More than 75% of cancer patients were reported to experience death and catastrophic payments within 1 year in Southeast Asia, especially those without health insurance (21). Other studies performed in China found that CHE occurred in 78.1% of the families of lung cancer patients and 66.28% of the families of breast cancer patients, representing ultra-high OOP expenditures on healthcare in these patient groups (22, 23). Health insurance is thought to be effective against OOP spending, but large disparities exist in the benefit packages between regions with different levels of economic development (24). The Urban Employee Basic Medical Insurance (UEBMI) provides the highest reimbursement cap of the available governmental options.

Reliable predictors of healthcare utilization and CHE are needed to provide further insight for policy makers. This is particularly important now as the cancer spectrum in China transitions from a developing country to a developed one due to its dynamic socio-economic development (25). Efficient cancer control and management systems should be designed and optimized to fit this transition. This work utilized a nationally representative database to investigate factors pertinent to cancer incidence, health service utilization, and CHE among cancer patients in China. Findings may be useful in the development and refinement of individualized and targeted policies to relieve the cancer burden in China.

## Methods

### Data source

Data used for the current study were derived from the China Health and Retirement Longitudinal Study (CHARLS) in 2018. The CHARLS is a nationwide representative longitudinal survey performed by the National Development Institute of Peking University to serve the needs for scientific and policy research on aging issues by concentrating on Chinese people aged 45 and older and their families. CHARLS included variables related to the demographics, lifestyle habits, health status, health care, household income and consumption, and health insurance of both urban and rural residents (26). CHARLS included 150 districts and 450 rural/urban communities in 28 provinces with a multiple-stage stratified random sampling method to ensure a nationally representative sample. A total of 19,507 respondents were involved in the 2018 wave, which included 392 individuals who were clinically diagnosed with cancer. We eliminated respondents with missing health information or household consumption data, leaving a sample of 18,968 individuals (388 cancer patients).

### Indicators

In CHARLS, all respondents were interviewed if they self-identified as clinically diagnosed with cancer through the following question: “Have you been diagnosed with cancer or malignant tumor (excluding minor skin cancers) by a doctor?” The cancer site was recorded. Respondents with minor skin cancers were excluded from the questionnaire as their cancer survivorship care demands tended to be relatively mild (18).

The number of monthly outpatient visits and annual inpatient days were used to measure health care utilization among the cancer patients. In CHARLS, individuals self-reported their utilization of outpatient and inpatient care through the questions: “How many times did you visit/been visited by medical facilities for outpatient care during the last month?” and “How many days did you spend in the hospital during the past year?” CHARLS gathered information on self-reported medical expenditure and total household expenditure for each family. Medical expenditure referred to the patient's outpatient expenses over the past month multiplied by 12 and inpatient expenses over the past year, including OOP expenditure and the portion reimbursed by health insurance. Regarding the total annual household expenditure, we multiplied monthly household expenses on rent, food, clothing, communication, water and electricity, fuel, services, education, traveling, entertainment, beauty, donations, daily necessities, and healthcare by 12.

Annual per-capita household consumption expenditure was adopted to gauge socioeconomic status as household consumption captured the actual living situation of each household (8). We identified five socioeconomic groups using quintiles of annual per-capita household consumption expenditure. Socioeconomic quintiles were established within each county or district and then combined across all sampled counties and districts to reflect the variable level of economic development across the targeted areas.

We defined an OOP payment on health care to be catastrophic if it surpassed 40% of the household's affordability, defined as non-food household consumption spending (8). Based on previous studies, CHE is a binary variable (8). We determined if CHE occurred by calculating the *Ei*:


$$Ei=\{\begin{array}{c} 0,\;\frac{oop}{xi-f\left(x\right)}\leq Z\\ 1,\frac{oop}{xi-f(x)}>Z \end{array}$$

Where *oop* denotes direct medical expenses, deducting reimbursement by health insurance, *i* represents various households, *x* is total household consumption expenditure, *f*(*x*) is food expenditure, and *Z* is the CHE threshold, which is set at 40%.

### Variables

We considered the following individual-level and household-level variables to be covariates: gender (male and female); age (45~65 and >65 years); household registration (agriculture and non-agriculture); education level (primary school and below, secondary school, and college and above); marital status (married, unmarried, divorced, and widowed); employment status (employed, unemployed, jobless, and retired); physical examination (yes vs. no); family physician (contracted vs. non-contracted); impoverishment status (impoverished vs. non-impoverished); household size (1~2 and ≥3 members); sleep duration; number of chronic diseases; basic health insurance (uninsured, UEBMI, URRBMI, URBMI, NRCMS and the other); socioeconomic groups; and geographic region (east, central, west and northeast).

### Statistical analysis

The sociodemographic characteristics of cancer patients were described using frequencies and percentages of categorical variables (e.g., education level, marital status, work status, health insurance, and region). A binary logistic regression model was created to identify the risks of cancer among all participants and CHE in the households of cancer patients. A negative binomial regression analysis was utilized to investigate correlations between demographic and socioeconomic variables and the number of outpatient visits and inpatient days among cancer patients. Odds ratios (OR) and incidence rate ratios (IRR) were quantified to measure the degree of correlations between each contributor and the dependent variables. A sensitivity analysis was performed to investigate the relationship between socioeconomic status quintiles and CHE exposure using the World Bank's various definitions of CHE thresholds, which were computed as OOP expenditures on healthcare of 25 and 40% of non-food household consumption expenditures (27). All statistical analyses were performed using SPSS V.26.0. A two-tailed *P* < 0.05 was considered significant.

## Results

### Sociodemographic characteristics of cancer patients

The descriptive sociodemographic statistics of 388 participants with clinically-diagnosed cancer in 2018 are shown in Table 1. A total of 153 (39.4%) respondents were male and 235 (60.6%) were female. Mean age was 63.9 years, with 214 (55.2%) participants 45–65 years old and 174 (44.8%) over 65 years old. Most participants were from rural areas (67.8% of total) and had only primary education or below (67.0% of total). Three hundred thirty (85.1%) participants were married, and 146 (37.6%) were employed. Regarding household size, 246 (63.4%) households with cancer patients had less than three people. The majority (372, 95.9%) of participants were enrolled in at least one type of public health insurance, with 221 (57.0%) participating in the NRCMS. A plurality (146, 37.6%) of participants were from eastern China, followed by its central (29.6%), western (26.8%), and northeastern (5.9%) regions.

**Table 1:** Sociodemographic characteristics of cancer patients in China, 2018.

**Sociodemographic characteristics**	**Number**	**Percent (%)**
**Gender**		
Male	153	39.4
Female	235	60.6
**Age (years)**		
45~65	214	55.2
> 65	174	44.8
**Household registration**		
Agriculture	263	67.8
Non-agriculture	125	32.2
**Education level**		
Primary school and below	260	67.0
Secondary school	106	27.3
College and above	22	5.7
**Marital status**		
Married	330	85.1
Rest 1	58	14.9
**Employment status**		
Employed	146	37.6
Rest 2	242	62.4
**Household size**		
1~2	246	63.4
≥3	142	36.6
**Health insurance**		
None	16	4.1
UEBMI	84	21.6
URRBMI	42	10.8
URBMI	15	3.9
NRCMS	221	57.0
Other†	10	2.6
**Region**		
West	104	26.8
Northeast	23	5.9
Central	115	29.6
East	146	37.6

Rest 1 denotes unmarried, divorced, and widowed; Rest 2 stands for unemployed, jobless, and retired; UEBMI, Urban Employee Basic Medical Insurance; URRBMI, Urban and Rural Residents Basic Medical Insurance; URBMI, Urban Resident Basic Medical Insurance; NRCMS, New Rural Cooperative Medical Scheme; Other† represents government medical insurance.

### Cancer incidence and associated factors

Overall cancer incidence among Chinese adults aged 45 and older in 2018 was 2.05% (388 of 18,968). Participants in higher socioeconomic quintiles had a higher likelihood of reporting a cancer diagnosis compared than those with the lowest socioeconomic status [Quintile 4: OR = 2.46, 95% CI 1.69–3.59; Quintile 5 (highest): OR = 3.04, 95% CI 2.08–4.42]. The likelihood of cancer was higher among participants who lived in eastern China than those in western China (OR = 1.54, 95% CI 1.19–2.00). Compared to employed participants, those who were unemployed, jobless, or retired had a greater incidence of cancer (OR = 2.57, 95% CI 2.04–3.25). Compared with participants who had not had a physical examination, those who obtained a medical check-up had a higher probability of being diagnosed with cancer (OR = 1.32, 95% CI 1.07–1.63). The prevalence of cancer decreased among participants who slept longer (OR = 0.90, 95% CI 0.86–0.95), received a secondary education (OR = 0.76, 95% CI 0.58–0.98 compared with those who had a primary education or less), and were unmarried, divorced, or widowed (OR = 0.72, 95% CI 0.54–0.98, compared to those who were married) (Table 2).

**Table 2:** Cancer determinants among people aged 45 years and older in China, 2018.

**Variables**	**Odds ratio (95% CI)**	***P*** **-value**
**Gender (Ref**. **=** **male)**		
Female	1.17 (0.94, 1.46)	0.147
**Age (Ref**. **=** **45~65 years)**		
> 65 years	0.98 (0.77, 1.25)	0.869
**Household registration (Ref**. **=** **agriculture)**		
Non-agriculture	1.17 (0.80, 1.71)	0.407
**Education level (Ref**. **=** **primary school and below)**		
Secondary school	0.76 (0.58, 0.98)	**0.037**
College and above	0.66 (0.39, 1.10)	0.107
**Marital status (Ref**. **=** **married)**		
Rest 1	0.72 (0.54, 0.98)	**0.036**
**Employment status (Ref**. **=** **employed)**		
Rest 2	2.57 (2.04, 3.25)	**<** **0.001**
**Sleep duration**	0.90 (0.86, 0.95)	**<** **0.001**
**Physical examination (Ref**. **=** **no)**		
Yes	1.32 (1.07, 1.63)	**0.010**
**Family physician (Ref**. **=** **no)**		
Yes	0.64 (0.35, 1.18)	0.151
**Impoverished (Ref**. **=** **yes)**		
Non-impoverished	0.84 (0.55, 1.27)	0.400
**Household size (Ref**. **=** **1~2)**		
≥3	0.97 (0.78, 1.21)	0.810
**Basic health insurance (Ref**. **=** **uninsured)**		
UEBMI	0.84 (0.46, 1.55)	0.584
URRBMI	0.83 (0.46, 1.50)	0.537
URBMI	0.53 (0.25, 1.14)	0.103
NRCMS	0.91 (0.54, 1.55)	0.737
Other†	1.23 (0.51, 2.97)	0.643
**Socioeconomic group (Ref**. **=** **lowest)**		
Quintile 2	1.38 (0.92, 2.07)	0.123
Quintile 3	1.21 (0.79, 1.83)	0.378
Quintile 4	2.46 (1.69, 3.59)	**<** **0.001**
Quintile 5 (highest)	3.04 (2.08, 4.42)	**<** **0.001**
**Region (Ref**. **=** **west)**		
Northeast	1.04 (0.66, 1.66)	0.855
Central	1.29 (0.99, 1.69)	0.064
East	1.54 (1.19, 2.00)	**0.001**

Rest 1 denotes unmarried, divorced, and widowed; Rest 2 stands for unemployed, jobless, and retired; UEBMI, Urban Employee Basic Medical Insurance; URRBMI, Urban and Rural Residents Basic Medical Insurance; URBMI, Urban Resident Basic Medical Insurance; NRCMS, New Rural Cooperative Medical Scheme; Other† represents government medical insurance.

### Factors associated with health care utilization

Factors associated with health care utilization and CHE among cancer patients are presented in Table 3. An increasing number of non-communicable diseases (IRR = 1.20, 95% CI 1.10–1.31) and contracting a family physician (IRR = 2.72, 95% CI 1.01–7.35) were associated with more frequent outpatient visits. Compared with those with a primary education and below, patients with college education and above reported more frequent outpatient visits (IRR = 5.78, 95% CI 2.56–13.02) but fewer inpatient hospital days (IRR = 0.37, 95% CI 0.20–0.67). Fewer inpatient hospital days were also found to be positively associated with patients who were female (IRR = 0.55, 95% CI 0.43–0.71), non-agricultural (IRR = 0.25, 95% CI 0.14–0.44), unmarried, divorced, or widowed patients (IRR = 0.57, 95% CI 0.40–0.82), and from eastern China (IRR = 0.68, 95% CI 0.49–0.93). Fewer outpatient visits were also positively associated with patients in families of more than 2 persons (IRR = 0.53, 95% CI 0.34–0.83) and those enrolled in URRBMI (IRR = 0.30, 95% CI 0.10–0.86). Longer inpatient hospital stays were positively associated with patients who were unemployed, jobless, or retired (IRR = 3.37, 95% CI 2.56–4.44), contracted with a family physician (IRR = 2.38, 95% CI 1.18–4.77), in a household of more than two members (IRR = 1.51, 95% CI 1.16–1.96), and enrolled in UEBMI (IRR = 4.02, 95% CI 1.91–8.46), URRBMI (IRR = 3.08, 95% CI 1.46–6.49). The likelihood of inpatient service use decreased substantially with economic status. The number of inpatient hospital days reported among patients in other socioeconomic quintiles were 0.23 (IRR = 0.23, 95% CI 0.16–0.33), 0.11 (IRR = 0.11, 95% CI 0.08–0.16), 0.10 (IRR = 0.10, 95% CI 0.06–0.15), and 0.07 (IRR = 0.07, 95% CI 0.05–0.11) times as many as those in the highest socioeconomic quintile, respectively.

**Table 3:** Factors associated with health care utilization and catastrophic health expenditure among cancer patients in China, 2018.

	**Number of outpatient visits**		**Inpatient hospital days**		**Catastrophic health expenditure**	
	**Incidence rate ratio (95% CI)**	***P*** **-value**	**Incidence rate ratio (95% CI)**	***P*** **-value**	**Odds ratio (95% CI)**	***P*** **-value**
**Number of non-communicable diseases**	1.20 (1.10, 1.31)	**<** **0.001**	1.06 (1.00, 1.12)	0.057	1.10 (0.98, 1.24)	0.105
**Gender (Ref**. **=** **male)**						
Female	1.27 (0.84, 1.92)	0.265	0.55 (0.43, 0.71)	**<** **0.001**	0.65 (0.39, 1.08)	0.094
**Age (Ref**. **=** **45~65 years)**						
> 65 years	1.58 (1.01, 2.47)	**0.045**	1.20 (0.90, 1.60)	0.206	1.46 (0.86, 2.47)	0.161
**Household registration (Ref**. **=** **agriculture)**						
Non-agriculture	0.64 (0.28, 1.48)	0.294	0.25 (0.14, 0.44)	**<** **0.001**	0.46 (0.16, 1.20)	0.110
**Education level (Ref**. **=** **primary school and below)**						
Secondary school	1.29 (0.79, 2.13)	0.312	0.75 (0.55, 1.03)	0.075	1.24 (0.68, 2.26)	0.473
College and above	5.78 (2.56, 13.02)	**<** **0.001**	0.37 (0.20, 0.67)	**0.001**	0.52 (0.15, 1.78)	0.298
**Marital status (Ref**. **=** **married)**						
Rest 1	0.68 (0.38, 1.22)	0.194	0.57 (0.40, 0.82)	**0.003**	1.20 (0.60, 2.39)	0.613
**Employment status (Ref**. **=** **employed)**						
Rest 2	1.40 (0.90, 2.18)	0.138	3.37 (2.56, 4.44)	**<** **0.001**	2.68 (1.59, 4.53)	**<** **0.001**
**Physical examination (Ref**. **=** **no)**						
Yes	0.95 (0.63, 1.44)	0.813	0.81 (0.62, 1.04)	0.098	0.80 (0.49, 1.31)	0.376
**Family physician (Ref**. **=** **no)**						
Yes	2.72 (1.01, 7.35)	**0.048**	2.38 (1.18, 4.77)	**0.015**	0.92 (0.23, 3.71)	0.910
**Impoverished (Ref**. **=** **yes)**						
Non**-**impoverished	1.95 (0.77, 4.93)	0.159	1.31 (0.82, 2.10)	0.265	0.43 (0.16, 1.17)	0.099
**Household size (Ref**. **=** **1~2)**						
≥3	0.53 (0.34, 0.83)	**0.006**	1.51 (1.16, 1.96)	**0.002**	0.52 (0.32, 0.86)	**0.010**
**Health insurance (Ref**. **=** **uninsured)**						
UEBMI	0.46 (0.16, 1.30)	0.143	4.02 (1.91, 8.46)	**<** **0.001**	0.38 (0.10, 1.43)	0.153
URRBMI	0.30 (0.10, 0.86)	**0.025**	3.08 (1.46, 6.49)	**0.003**	1.61 (0.40, 6.46)	0.501
URBMI	0.42 (0.09, 1.99)	0.277	1.93 (0.74, 5.04)	0.181	0.80 (0.15, 4.39)	0.802
NRCMS	0.55 (0.23, 1.32)	0.181	1.21 (0.63, 2.32)	0.564	0.64 (0.19, 2.18)	0.472
Other†	0.21 (0.04, 1.09)	0.063	1.90 (0.64, 5.58)	0.246	0.60 (0.09, 3.86)	0.588
**Socioeconomic group (Ref**. **=** **highest)**						
Quintile 2	0.60 (0.35, 1.05)	0.074	0.23 (0.16, 0.33)	**<** **0.001**	0.35 (0.17, 0.74)	**0.006**
Quintile 3	0.36 (0.20, 0.66)	**<** **0.001**	0.11 (0.08, 0.16)	**<** **0.001**	0.39 (0.19, 0.81)	**0.011**
Quintile 4	0.56 (0.30, 1.05)	0.071	0.10 (0.06, 0.15)	**<** **0.001**	0.36 (0.16, 0.81)	**0.013**
Quintile 5 (lowest)	0.57 (0.31, 1.05)	0.069	0.07 (0.05, 0.11)	**<** **0.001**	0.32 (0.14, 0.72)	**0.006**
**Region (Ref**. **=** **west)**						
Northeast	0.33 (0.10, 1.09)	0.068	1.74 (0.98, 3.09)	0.059	0.63 (0.22, 1.81)	0.391
Central	0.79 (0.48, 1.31)	0.363	1.13 (0.80, 1.61)	0.486	0.80 (0.43, 1.50)	0.488
East	0.94 (0.59, 1.49)	0.789	0.68 (0.49, 0.93)	**0.017**	0.67 (0.37, 1.22)	0.192

Rest 1 denotes unmarried, divorced, and widowed; Rest 2 stands for unemployed, jobless, and retired; UEBMI, Urban Employee Basic Medical Insurance; URRBMI, Urban and Rural Residents Basic Medical Insurance; URBMI, Urban Resident Basic Medical Insurance; NRCMS, New Rural Cooperative Medical Scheme; Other† represents government medical insurance.

### CHE incidence and associated factors

50.1% of households with cancer patients were considered to have CHE when a 40% threshold was used. The prevalence of CHE was greater amongst patients who were unemployed, jobless, or retired (OR = 2.68, 95% CI 1.59–4.53) than those who were employed. Compared with households of fewer than three members, a larger family size was protective from CHE (OR = 0.52, 95% CI 0.32–0.86). Further, patients in a lower socioeconomic class had a smaller probability of reporting CHE than those in a higher socioeconomic class. There was no discernible relationship between health insurance and CHE (Table 3). A sensitivity analysis suggested that there was a non-significant correlation between economic status and CHE when the World Bank's definition of CHE at a 25% threshold was used (Supplementary Table S1), which was inconsistent with the base case analysis. In addition, the odds of CHE were lower in non-agricultural households (OR = 0.33, 95% CI 0.12–0.92, compared with agricultural households).

## Discussion

This study identifies factors potentially associated with health care utilization and CHE among Chinese cancer patients using a nationwide representative longitudinal survey of the middle-aged and elderly population. These findings provide insights into health service utilization and economic burden of cancer patients. The socioeconomic characteristics (public health insurance and household consumption level) of the patients and their families appear to have significant influence on cancer care. CHE was reported by approximately half of the families of cancer patients, with it occurring more prevalently in higher-income families than in lower-income ones. We also found that cancer was more commonly reported by people with a lower educational level, who were married, unemployed, jobless, or retired, who self-reported a shorter sleep duration, who underwent regular physical examinations, who were in a higher socioeconomic group, and who resided in eastern China.

We observed that cancer was more commonly reported by respondents who were in a higher socioeconomic category. This may be because respondents living in higher-income families had greater access to better healthcare delivery and better wellness education, and therefore were more likely to have an underlying cancer diagnosed than those who lived in lower-income families (28). Individuals from the eastern region reported an increased prevalence of cancer, which likely reflects under-reporting in the western region due to the relative shortage of health resources and accessibility (29). In agreement with a previous study, sleeping for fewer hours was correlated with cancer in Chinese people, which may be the result of physiologic mechanisms (30). Given the cross-sectional nature of our data, we were only able to establish a longitudinal association between sleep duration and cancer incidence.

Health service use by cancer patients in our study was primarily driven by gender, age, household registration, education level, marital status, employment status, physical examination, family physician, household size, health insurance, per-capita household consumption (socioeconomic status), and the number of chronic non-communicable diseases. We observed that the utilization of outpatient care increased as the number of chronic non-communicable diseases increased. This is likely due to the fact that cancer patients with additional diseases are more likely to have complications, and the subsequent demand for intensified care and coordinated treatment could increase outpatient visits (31). This association has been well documented in other studies on multimorbidity (31–33). Also, in agreement with other studies, cancer patients in higher socioeconomic groups were more likely to access and utilize both outpatient and inpatient services (28, 34, 35), exposing inequalities in cancer care. If the economic struggles resulting from cancer are not addressed, its negative impact on healthcare access and utilization may contribute to the deteriorated health status of patients in lower socioeconomic categories and increased cancer mortality (36). In contrast, patients with a higher socioeconomic status had a better prognosis, which may be associated with a higher level of care or even over-medication (17). Our results also found that patients who contracted with a family physician had more frequent outpatient visits and longer inpatient stays. A plausible explanation might be that contracting with a family physician is associated with higher income levels, which could enhance patient disease awareness and treatment compliance (37). However, family physicians serve as the gatekeepers to health care, aiming to prevent chronic diseases by intervening in disease-related lifestyle behaviors in addition to delivering primary health care services that can efficiently lower the hospitalization and mortality of patients and mitigate the burden of chronic disease in China (37). The effect of family physician contracting on the outpatient and inpatient services that were utilized by cancer patients remains for consideration.

We found that individuals with a better educational background utilized more outpatient services but fewer inpatient services. There are several possible reasons for this discrepancy. First, individuals with higher levels of education tend to rank higher in socioeconomic status and capture more appropriate pathways of care (38), such as well check-ups, consultations, and outpatient visits, permitting diseases to be identified at an early stage. Second, populations with lower levels of education may have inadequate knowledge of cancer-related symptoms and signs, so if they took these lightly or lacked illness awareness, they would not approach a clinician for a timely diagnosis (39). The observation that individuals with lower levels of education are at higher risk of cancers of the esophagus, stomach, rectum, rectosigmoid colon, liver, pancreas, lung, kidney, and urinary tract has been previously reported (40), and patients with these cancers often require radiotherapy, chemotherapy, or more intensive treatment (41), requiring a longer inpatient stay.

Social health insurance plans are designed to promote nationwide access to healthcare. Our results showed that patients enrolled in UEBMI and URRBMI reported significantly longer inpatient stays compared with non-enrolled patients, whereas URBMI and NCRMS did not influence this outcome. An explanation for this observation is that fewer inpatient services were available to people who were covered by URBMI and NCRMS due to restricted benefits packages and reduced coverage compared with other schemes (24). Cancer patients enrolled in UEBMI were more likely to spend more time in the hospital, suggesting that UEBMI is superior to URRBMI in terms of reimbursement level. Regarding outpatient visits, our findings showed that health insurance plans seemed to have minimal impact on the decision to utilize outpatient services except for the URRBMI, whose members had fewer outpatient visits compared with uninsured patients. A previous study reported that Chinese people tended to favor inpatient services over outpatient services regardless insurance type (42), and that cancer patients might have a stronger preference toward hospitalization due to their unique treatment needs.

Our study reported that the prevalence of families with cancer patients that experienced CHE was 50.1% at a 40% threshold. Compared with other cancer studies that used the same definition and threshold of CHE, our study of Chinese showed a higher prevalence of CHE than Iran (13.77%) (43) and Malaysia (47.8%) (44), which might be attributed to the disparities in the income levels and health insurance packages between these countries. The overall prevalence of CHE among the general Chinese population was only 8.94% in 2016 in China (45). The significant discrepancy indicates that OOP spending on cancer care imposes a substantial financial burden on more than half of Chinese families with cancer patients. Moreover, the calculation of OOP spending we used only captured the direct expenses of cancer care, and our statistic is therefore conservative given the exclusion of indirect expenses such as transport and accommodation. Our findings also suggest that a large household size could shelter some families with cancer patients against CHE. One potential hypothesis for this is that family members would care for each other and provide both material and psychological assistance to those with severe diseases. A larger household size may also represent increased household consumption and income, and therefore may be a protective factor when income exceeds consumption.

While it is generally acknowledged that better-off families were more capable of coping with healthcare spending than poorer families (21), our study found that households with cancer patients that were at a higher socioeconomic level had a higher probability of experiencing CHE. There are several possible explanations for this inconsistency. First, most cancer patients with a low economic status in our study were from agricultural families. They were less likely to purchase health services due to their inadequate health knowledge of the incidence of cancer and inefficient allocation of health resources to rural regions, which may reduce the incidence of CHE (39, 46). Secondly, cancer therapy is highly expensive due to the need for repetitive hospitalizations, multiple consultations, advanced laboratory examinations, chemotherapy, rare and costly drugs, surgery and radiation therapy, and other essential care (47). Cancer patients with a low economic status might therefore forgo therapy on account of the high OOP payments and the potential impact of their care on the livelihoods of other family members, making it possible for families to avert CHE but resulting in worse health outcomes. In contrast, people in higher-income classes require greater absolute levels of spending than people in lower-income classes to trigger the so-called CHE threshold. Our results suggest that higher OOP expenses could be reflective of receiving more intensive and expensive health-care services (36), or the purchase of high-quality services from private facilities by bypassing the inconvenience of public facilities. Private healthcare services are generally more expensive than public services because they are primarily driven by the demand of better-off individuals for high-quality services (48). Finally, while the improved survival of better-off cancer patients was the result of proactive therapy, post-cancer care requires sustained financial support (17), which may increase the risk of CHE. We also found that CHE had no significant association with health insurance, indicating that public health insurance failed reduce the financial risks posed by cancer to the patient's family.

Our findings add to the body of evidence that supports the development of targeted policies and strategies to address China's growing cancer burden. The occurrence of cancer among middle-aged and elderly patients is significantly affected by social variables such as education level, marital status, employment and socioeconomic status, and geography, which involve multiple societal domains. Contemporary public health strategies require cross-sectoral collaboration to improve social determinants of health and implement effective prevention strategies that target high-risk populations (49). We also observed a significant association between health care utilization and the socioeconomic characteristics of cancer patients. Policymakers should take the socioeconomic burdens experienced by cancer patients into consideration when formulating practice guidelines on cancer management so as to facilitate efficient and individualized treatment solutions. For example, a nationwide representative cancer registry with enhanced quality and targeting will facilitate the identification of treatment priorities and care utilization among Chinese cancer patients (50). To alleviate the burden of providing cancer care and improve disease prognosis, the government needs to raise screening awareness among targeted vulnerable populations and enhance the likelihood of detecting cancer at an early stage. A cancer screening and early detection network is present in 31 provinces of China as of 2015, but whole population screenings are not offered except for breast and cervical cancer (51). OOP spending is the most significant determinant of catastrophic expenditure. In China, the high OOP expenditures on healthcare may be due to fee-for-service payment mechanisms and the failure of public health insurance to bear financial risks (52). Cancer care payment model reform is required to improve the financial burden that cancer treatment places on Chinese families. A patient-centered and value-oriented alternative payment model for oncology is recommended due to its significant associations with improved cancer care quality and reduced resource use and care costs (53). China has almost achieved universal health insurance coverage, yet catastrophic payments for cancer patients remain too high. This may be due to the restrictions of current benefit packages and the small scale of medical aid (54). Future health insurance schemes should strengthen financial protection for Chinese cancer patients in a targeted manner.

Our study had several limitations. First, given that cancer diagnosis was self-reported, the incidence of cancer in China was potentially underestimated. This assumption is even more profound in vulnerable populations who are less likely to be diagnosed with cancer at an early stage. Second, health service use and health payments among cancer patients were also self-reported. These are also prone to underestimation, especially among older adults and those with a lower education level. Third, our sample size was rather small for this type of study. A larger sample should be used in future work. Given that many of our findings indicate that better-off families were less likely to experience CHE (45), future studies should use other indicators of socioeconomic groups beyond socioeconomic status quintiles. Future work should also take into consideration variables related to mental health because of its significant impact on care-seeking behaviors (55). Finally, this research only recruited Chinese people aged 45 years and older. Future studies should consider the impact of cancer on younger cohorts.

## Conclusion

The socioeconomic characteristics of cancer patients had a considerable impact on their healthcare utilization, especially health insurance and socioeconomic status. OOP spending on cancer care imposed a substantial financial burden on more than half of Chinese households with cancer patients, particularly those from better-off households. Public health insurance failed to reduce the financial risks posed to cancer patients' families. Individualized and targeted guidelines for cancer management, strategic purchasing models in cancer care, and social health insurance with expanded benefit packages are crucial to easing the burden of cancer care in China.

## Data availability statement

The original contributions presented in the study are included in the article/Supplementary material, further inquiries can be directed to the corresponding author.

## Author contributions

PD led the study including the interpretation of the results and manuscript drafting. LS and MC conceived and supervised the study. YF contributed to data analysis and drafting of the manuscript. All authors reviewed and approved the final manuscript.

## Funding

This study was funded by the National Natural Science Foundation of China (Grant Numbers: 71874086 and 72174093) and the Postgraduate Research and Practice Innovation Program of Jiangsu Province (Grant Number: KYCX21_1561).

## Conflict of interest

The authors declare that the research was conducted in the absence of any commercial or financial relationships that could be construed as a potential conflict of interest.

## Publisher's note

All claims expressed in this article are solely those of the authors and do not necessarily represent those of their affiliated organizations, or those of the publisher, the editors and the reviewers. Any product that may be evaluated in this article, or claim that may be made by its manufacturer, is not guaranteed or endorsed by the publisher.
