# Supplementary material for: Factors associated with health care utilization and catastrophic health expenditure among cancer patients in China: Evidence from the China health and retirement longitudinal study
Source: Front Public Health. 2022 Nov 10;10:943271. doi: 10.3389/fpubh.2022.943271 (PMC9684646; doi:10.3389/fpubh.2022.943271)
Supplement: Supplementary file 1 [file Table_1.DOCX]

| **Supplementary Table** | | |
| --- | --- | --- |
| **Supplementary Table S1 \|** Factors Associated with Catastrophic Health Expenditure at a 25% threshold among Cancer Patients in China, 2018 | | |
|  | **Odds ratio (95% CI)** | ***P-*value** |
| **Number of non-communicable diseases** | 1.07 (0.95, 1.21) | 0.270 |
| **Gender (Ref. = male)** |  |  |
| Female | 0.68 (0.41, 1.13) | 0.137 |
| **Age (Ref. = 45~65 years)** |  |  |
| > 65 years | 1.28 (0.75, 2.18) | 0.369 |
| **Household registration (Ref. = agriculture)** |  |  |
| Non-agriculture | 0.33 (0.12, 0.92) | **0.035** |
| **Educational level (Ref. = primary school and below)** |  |  |
| Secondary school | 1.05 (0.58, 1.92) | 0.870 |
| College and above | 0.78 (0.26, 2.29) | 0.647 |
| **Marital status (Ref. = married)** |  |  |
| Rest 1 | 1.19 (0.58, 2.44) | 0.637 |
| **Employment status (Ref. = employed)** |  |  |
| Rest 2 | 3.03 (1.79, 5.14) | **< 0.001** |
| **Physical examination (Ref. = no)** |  |  |
| Yes | 0.82 (0.50, 1.35) | 0.441 |
| **Family physician (Ref. = no)** |  |  |
| Yes | 0.82 (0.20, 3.45) | 0.791 |
| **Impoverished (Ref. = yes)** |  |  |
| Non**-**impoverished | 0.32 (0.10, 1.01) | 0.052 |
| **Household size (Ref. = 1~2)** |  |  |
| ≥3 | 0.46 (0.28, 0.75) | **0.002** |
| **Health insurance (Ref. = uninsured)** |  |  |
| UEBMI | 0.62 (0.16, 2.42) | 0.496 |
| URRBMI | 1.45 (0.34, 6.22) | 0.615 |
| URBMI | 0.66 (0.12, 3.63) | 0.631 |
| NRCMS | 0.61 (0.17, 2.17) | 0.442 |
| Other† | 0.86 (0.13, 5.77) | 0.879 |
| **Socioeconomic group (Ref. = highest)** |  |  |
| Quintile 2 | 0.66 (0.31, 1.40) | 0.281 |
| Quintile 3 | 0.54 (0.26, 1.14) | 0.107 |
| Quintile 4 | 0.54 (0.24, 1.23) | 0.140 |
| Quintile 5 (lowest) | 0.44 (0.19, 1.00) | 0.051 |
| **Region (Ref. = west)** |  |  |
| Northeast | 0.71 (0.26, 1.97) | 0.511 |
| Central | 0.88 (0.46, 1.66) | 0.688 |
| East | 0.64 (0.35, 1.17) | 0.149 |
| *Rest 1 denotes unmarried, divorced, and widowed; Rest 2 stands for unemployed, jobless, and retired; UEBMI, Urban Employee Basic Medical Insurance; URRBMI, Urban and Rural Residents Basic Medical Insurance; URBMI, Urban Resident Basic Medical Insurance; NRCMS, New Rural Cooperative Medical Scheme; Other† represents* g*overnment medical insurance.* | | |
